# Robot-Assisted Pancreaticoduodenectomy Using the Anterior Superior Mesenteric Artery-First Approach for Pancreatic Cancer

**DOI:** 10.1245/s10434-024-16305-6

**Published:** 2024-09-27

**Authors:** Kosei Takagi, Tomokazu Fuji, Kazuya Yasui, Motohiko Yamada, Takeyoshi Nishiyama, Yasuo Nagai, Noriyuki Kanehira, Toshiyoshi Fujiwara

**Affiliations:** Department of Gastroenterological Surgery, Okayama University Graduate School of Medicine, Dentistry, and Pharmaceutical Sciences, Okayama, Japan

**Keywords:** Robotic pancreaticoduodenectomy, Superior mesenteric artery approach, Pancreatic cancer

## Abstract

**Background:**

The superior mesenteric artery (SMA)-first approach for pancreatic cancer (PC) is common surgical technique in pancreaticoduodenectomy. To date, few studies have reported SMA-first approach in robot-assisted pancreaticoduodenectomy (RPD). Herein, we present the anterior SMA-first approach for PC during RPD.

**Patient and Method:**

A 75-year-old man with resectable PC underwent RPD after neoadjuvant chemotherapy. As pancreatic head tumor contacted with the superior mesenteric vein (SMV), the anterior SMA approach was applied. After the mesenteric Kocher maneuver, the jejunum was divided and the left side of the SMA was dissected. Subsequently, the anterior plane of the SMA was dissected. Following the division of branches from the mesenteric vessels, the SMA was taped, and the circumferential dissection around the SMA was performed to detach the pancreatic neck from the SMA completely. Finally, the dissection between the SMV and the tumor was performed under vascular control to remove the specimen.

**Conclusions:**

The anterior SMA-first approach can be optional in patients with PC undergoing RPD. This unique approach allows for the circumferential dissection around the SMA during RPD.

**Supplementary Information:**

The online version contains supplementary material available at 10.1245/s10434-024-16305-6.

The superior mesenteric artery (SMA)-first approach for pancreatic cancer (PC) is commonly used surgical technique during pancreaticoduodenectomy.^1^ However, few studies have reported SMA-first approach in robot-assisted pancreaticoduodenectomy (RPD).^2,3^ Herein, we present the anterior SMA-first approach for PC during RPD (supporting video 1).

## Case

A 75-year-old man with resectable PC underwent RPD after neoadjuvant chemotherapy of gemcitabine combined with S1. Preoperative computed tomography images demonstrated a 28-mm tumor at the pancreatic head contacting with the superior mesenteric vein (SMV).

## Surgical Technique

**Fig. 1:**
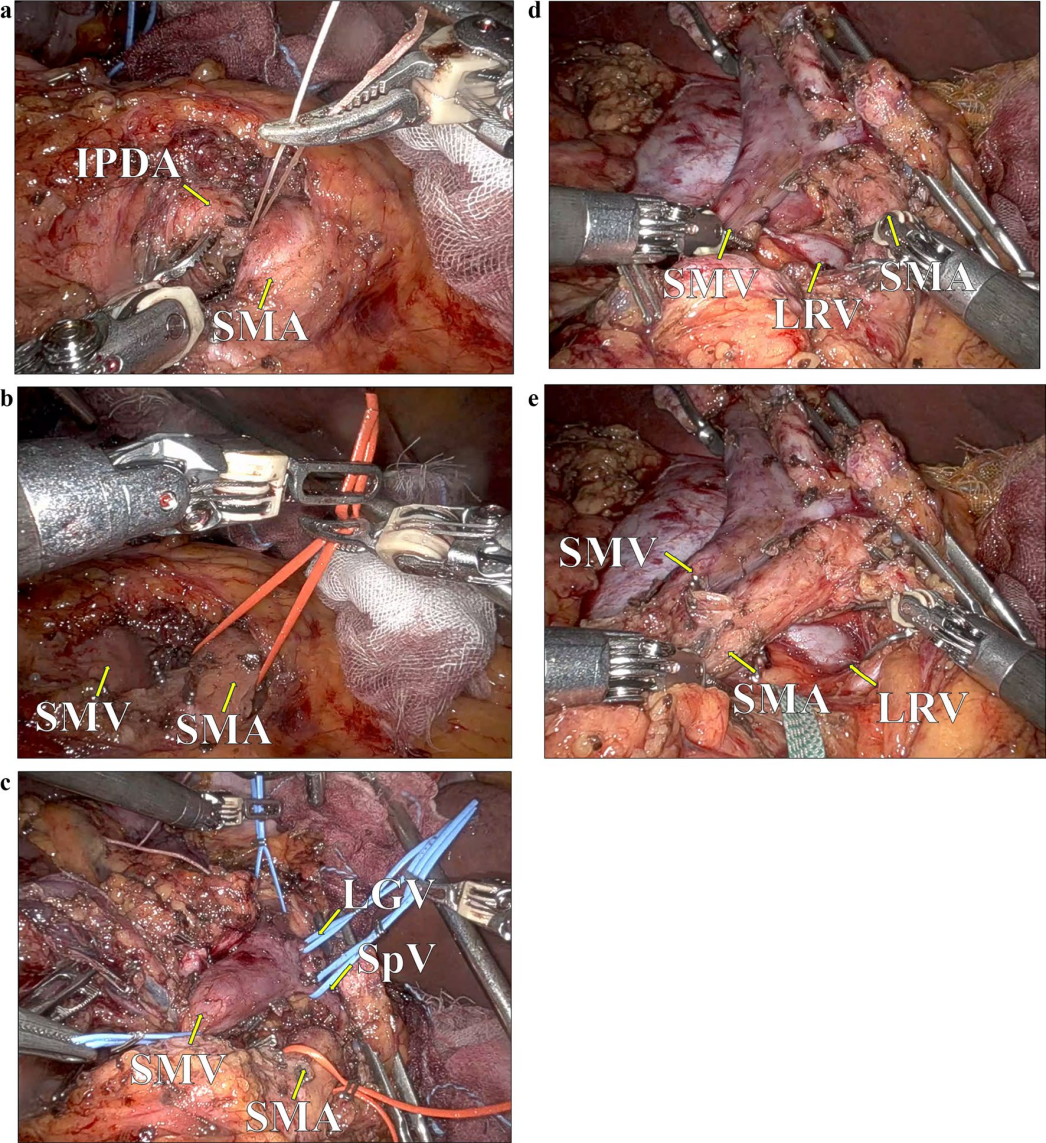
Anterior superior mesenteric artery (SMA)-first approach during robotic pancreaticoduodenectomy; **A** after dissecting the anterior plane of the SMA, the inferior pancreaticoduodenal artery was isolated and divided, **B** the SMA was encircled and taped, **C** for vascular control of the portal vein (PV) system, the superior mesenteric vein, PV, splenic vein, and left gastric vein were taped, **D**, **E** the resected specimen; *SMA* superior mesenteric artery, *SMV* superior mesenteric vein, *IPDA* inferior pancreaticoduodenal artery, *SpV* splenic vein, *LGV* left gastric vein, *LRV* left renal vein

After docking the robotic system (da Vinci Xi), the transverse colon was lifted cranially, and the mesenteric Kocher maneuver was introduced.^4^ The jejunum was divided using a stapler, and the mesojejunum was dissected along the first jejunum artery (J1A). The root of the J1A was divided, and the left and dorsal sides of the SMA was dissected along the neural plexus toward the root of the SMA. After repositioning the transverse colon and dividing the stomach, the anterior SMA approach was applied. Initially, the anterior plane of the SMA was dissected. Next, the right side of the SMA was also dissected, and the inferior pancreaticoduodenal artery (IPDA) was divided (Fig. 1A). Subsequently, the SMA was encircled and taped (Fig. 1B). For vascular control of the portal vein (PV) system, the SMV, PV, splenic vein, and coronary vein were taped, followed by the division of the pancreas on the SMA (Fig. 1C). The circumferential dissection around the SMA was performed in a cranial direction to detach the pancreatic neck from the SMA completely. During the dissection for right side of the SMA, proper retraction of the pancreatic head allowed to create a wide enough window for safe dissection. At this step, the specimen was connected only with the SMV. After clamping the SMV, PV, splenic vein, and coronary vein, the specimen was resected requiring no vascular reconstruction (Fig. 1D, E).

The operative time was 476 min, with an estimated blood loss of 50 mL. The patient was discharged on postoperative day 7 with good clinical course. The pathological findings revealed successful R0 resection.

## Discussion

The present study demonstrates the anterior SMA-first approach for PC in RPD. This approach has several advantages. Anterior SMA approach could facilitate better retroperitoneal dissection and no-touch technique with en bloc kocherization, but require early division of stomach and neck of pancreas.^1^ Using the anterior SMA approach, the circumferential dissection around the SMA can be performed in a caudal view during RPD. Moreover, it allows for complete detachment of the pancreatic neck from the SMA with the specimen connected with the SMV/PV. Therefore, this approach could also help in patients with borderline PC requiring the SMV/PV reconstruction. However, the weakness of this technique should be disclosed. Special caution should be paid to dissect dorsal branches from the mesenteric vessels such as IPDA. The dorsal side of the mesenteric vessels would be invisible in performing the anterior SMA approach. Therefore, injuries of dorsal branches from the mesenteric vessels could cause massive bleeding. To avoid serious injury of these vessels, the dissection around the dorsal side of the SMA can be performed from the left side. Using proper retraction of the pancreatic head, dorsal branches from the mesenteric vessels can be dissected from the right side as well. Another disadvantage would include the division of the stomach and pancreatic neck at an early stage to achieve adequate exposure of the SMA.^1^ Regarding the indication of the anterior SMA approach, this technique would not be effective for a tumor that is not contacting or infiltrating the mesenteric vessels. The current Brescia guidelines have suggested that the artery-first approach during MIPD should be tailored on a case-by-case basis by understanding each approach to the SMA dissection.^5^

## Conclusions

The anterior SMA-first approach can be safe and feasible in patients with PC undergoing RPD. This unique approach allows for the circumferential dissection around the SMA during RPD.

## Supplementary Information

Below is the link to the electronic supplementary material.

Video 1. Robotic pancreaticoduodenectomy using the anterior superior mesenteric artery first approach for pancreatic cancer (MP4 306270 kb)
